# Abstainers and Life Insurance

**Published:** 1889-04-06

**Authors:** 


### Editor's Letter-Box.

[Our Correspondents are reminded that prolixity is a great bar to publication, and that brevity of style and conciseness of statement greatly facilitate early insertion.]

### ABSTAINERS AND LIFE ASSURANCE: A REMINISCENCE.

The following communication has been forwarded by a correspondent in response to an article which appeared in *The Hospital* of February 16 on the subject of the longevity of total abstainers. It will be read with much interest by all who study the great social questions of the day:

"In an article in 'The Political Reviews for the Mid-Essex Parliamentary Division' for January, 1889, appears the following, extracted from a sketch of Mr. Robert Warner's life:

"About the year 1840 Mr. Robert Warner thought it would be prudent to insure his life, but having taken the risk of becoming an abstainer from intoxicating liquors, the office he proposed to wanted to charge him an extra rate, so questionable and risky a fact was teetotalism then considered, even by the directors and doctors of the leading life offices. Strong in his own convictions of the scientific soundness of total abstinence, he conferred with other like-minded men, and, as the result, a new society was started, and Mr. Warner became the first director and the first policy-holder of the society now known as the 'United Kingdom Temperance and General Provident Institution.' His first policy for £250 is still in existence, framed and glazed as a valuable relic, and Mr. Warner is still a director and the chairman of the institution, having in his own experience falsified the croakings of the early critics, being now in his seventy-fourth year.

"It is also interesting to note that, with growing faith in the institution, Mr. Warner took out several other policies, amounting altogether to £2,000, paying annual premiums of £44 9s. 3d.; and on the profit sharing system, by the reduction of the annual premiums, he now enjoys the receipt of an annuity from the office instead of having to pay any premiums, his successors being still entitled (as they would had he died immediately after the insurances were effected) to the full amount insured for. And now the institution is a great financial success having four million of money well invested, taking first rank with the insurance offices of the day. Not only so, but it has solved a great problem. It has two branches whose accounts are kept quite distinct—one for total abstainers, the other for non-abstainers, and the result has proved that the abstainers live longer than the non-abstainers, and so accumulate more money; it being entirely a mutual office, the abstainers earn much higher bonuses than the non-abstainers.

"Mr. Robert Warner is a Quaker, following an unbroken line, from the time of George Fox in 1665, at which time his ancestor, John Warner, was living. He was brought up at his father's country house at Hoddesdon, Herts; his father was one of the founders of the Peace Society. Mr. Warner is head of the firm of John Warner and Sons, of Cripplegate, hydraulic engineers and bellfounders, and is now in robust health."

